# Genome-Wide Identification of *MDH* Family Genes and Their Association with Salt Tolerance in Rice

**DOI:** 10.3390/plants11111498

**Published:** 2022-06-02

**Authors:** Yanhong Zhang, Yulong Wang, Xingming Sun, Jie Yuan, Zhiqiang Zhao, Jie Gao, Xiaorong Wen, Fusen Tang, Mintai Kang, Buhaliqem Abliz, Zhanying Zhang, Hongliang Zhang, Fengbin Wang, Zichao Li

**Affiliations:** 1 State Key Laboratory of Agrobiotechnology, China Agricultural University, Beijing 100193, China; zhangyanhong9527@163.com (Y.Z.); wangyulong0724@126.com (Y.W.); sungene@cau.edu.cn (X.S.); gaojie931103@163.com (J.G.); zhangzhanying@cau.edu.cn (Z.Z.); zhanghl@cau.edu.cn (H.Z.); 2 Beijing Key Laboratory of Crop Genetic Improvement, College of Agronomy and Biotechnology, China Agricultural University, Beijing 100193, China; 3 Xinjiang Key Laboratory of Crop Biotechnology, Institute of Nuclear Technology and Biotechnology, Xinjiang Academy of Agricultural Sciences, Urumqi 830091, China; yuanjie801023@163.com (J.Y.); zzq086@163.com (Z.Z.); halqam2006@163.com (B.A.); 4 Key Laboratory of Saline-Alkali Soil Improvement and Utilization (Saline-Alkali Land in Arid and Semi-Arid Regions), Ministry of Agriculture and Rural Affairs, Urumqi 830091, China; 5 Rice Experiment Station in Wensu, Xinjiang Academy of Agricultural Sciences, Wensu, Aksu 843100, China; wxrylj@126.com (X.W.); xjtangfusen@163.com (F.T.); shuidaokmt@126.com (M.K.); 6 Institute of Grain Crops, Xinjiang Academy of Agricultural Sciences, Urumqi 830091, China

**Keywords:** rice (*Oryza sativa* L.), *MDH* gene family, gene-based association study, salt stress

## Abstract

Malate dehydrogenase (MDH) is widely present in nature and regulates plant growth and development, as well as playing essential roles, especially in abiotic stress responses. Nevertheless, there is no comprehensive knowledge to date on *MDH* family members in rice. In this study, a total of 12 *MDH* members in rice were identified through genome-wide analysis and divided into three groups on the basis of their phylogenetic relationship and protein-conserved motifs. Evolutionary analysis showed that MDH proteins from rice, maize and wheat shared a close phylogenetic relationship, and the *MDH* family was conserved in the long-term process of domestication. We identified two segmental duplication events involving four genes, which could be the major force driving the expansion of the *OsMDH* family. The expression profile, cis-regulatory elements and qRT-PCR results of these genes revealed that a few *OsMDH* showed high tissue specificity, almost all of which had stress response elements in the promoter region, and ten *MDH* members were significantly induced by salt stress. Through gene-based association analysis, we found a significant correlation between salt tolerance at the seedling stage and the genetic variation of *OsMDH8.1* and *OsMDH12.1*. Additionally, we found that the polymorphism in the promoter region of *OsMDH8.1* might be related to the salt tolerance of rice. This study aimed to provide valuable information on the functional study of the rice *MDH* gene family related to salt stress response and revealed that *OsMDH8.1* might be an important gene for the cultivar improvement of salt tolerance in rice.

## 1. Introduction

Malate dehydrogenase (MDH) is a kind of oxidoreductase, which uses NAD(H)/NADP(H) as a cofactor to catalyze the reversible reaction of oxidative dehydrogenation from malic acid to oxaloacetate, mainly functioning in the cytoplasm, mitochondria, plastid and chloroplast of plants [1,2]. Many studies have shown that MDH extensively participates in energy metabolism, respiration and reactive oxygen metabolism, and plays an important role in stress resistance [3,4,5,6]. In *Arabidopsis* mutant *pdNAD-MDH*, the blocked physiological process of embryonic development can prevent endosperm development [7], and the mutant seeds can only reach the spherical stage and develop into tiny, shriveled seeds, so that no homozygous plants can be produced. In maize [8], *ZmMDH4* mainly catalyzes the transformation from oxaloacetate to malate, and the knockout of *ZmMDH4* leads to glycolytic metabolic transformation and a significant disruption of mitochondrial complex activity, thereby reducing starch content and producing small and opaque grains. In rice, *FLO16* encodes an NAD-dependent cytoplasmic malate dehydrogenase (CMDH), and the reduction in the ATP content of *flo16* mutant leads to a significant decrease in the activity of starch synthesis-related enzymes in rice seeds [9]. It has been reported that the activity of MDH will change under many abiotic stresses [5,10,11]. Researchers isolated the malate dehydrogenase gene *GhmMDH1* expressed in mitochondria from upland cotton, which plays a role in leaf respiration and phosphorus acquisition, as well as in plant and root growth under phosphorus deficiency conditions [5]. The SgMDH of *Stylosanthes* has a higher catalytic efficiency for OAA and NADH, but its catalytic efficiency for malate and NAD^+^ is relatively low, and *SgMDHs* in stigma can participate in the response to metal stress [11]. The overexpression of the *MDH* gene can promote the synthesis of organic acids in alfalfa and develop its resistance to aluminum [10]. Although the *MDH* gene has diverse functions and plays important roles in the growth and development of plants, to date, only two rice *MDH* genes have been reported [9,12]. Therefore, the function of *MDH* genes in rice needs to be further studied.

Salt damage is one of the major environmental factors affecting seed germination, plant growth, yield and quality of rice, which has already become a worldwide problem [13,14]. Rice growth inhibition due to salt stress manifests in morphological differences and the disorder of key enzymes in various metabolic pathways [13,15], which ultimately leads to cellular oxidation and nutrient depletion [16]. It was reported that the expression of *NADP-MDH* increased under salt stress in rice varieties with different salt tolerance, suggesting that *MDH* may play an important role in salt tolerance [13]. The expression of the NAD-dependent *MDH* gene in apple cytoplasm is positively correlated with growth and metabolic activity and plays a part in plant growth and salt stress response [4]. Under salt stress, the overexpression of *MdcyMDH* leads to a significantly higher reduction activity of cyMDH and chMDH and a higher oxidation activity of mMDH than wild-type plants [17]. The overexpression of *ZmNADP-MDH* leads to an increase in chlorophyll and protein content, and a reduction in the production of H_2_O_2_ and malondialdehyde via membrane lipid peroxidation [18]. The overexpression of *NADP-MDH* can maintain the oxidation deoxidation environment, which leads to greater salt stress tolerance [18]. *OsMDH1* was identified from mutant rice material, which reversely regulates salt tolerance by reducing vitamin B6 content [12]. In conclusion, *MDH* genes play important roles in abiotic stress resistance in different plants, especially salt stress resistance. However, the relationship between other *MDH* members in rice and salt tolerance is not clear.

In recent years, with the deepening of the whole genome sequence research, the genome sequence of many plants has been determined, which can be used to identify the *MDH* gene family at the DNA, amino acid and protein level. To date, *MDH* gene families have been identified based on the whole genomes from different plant species, including *Arabidopsis thaliana* [7,19], *Stylosanthes guianensis* (stylo) [11], apple [20], poplar (*Populus trichocarpa*) [21] and cotton [22]. For example, the *Arabidopsis* genome encoded nine isoforms of *MDH*, including one *NADP-MDH* and eight *NAD-MDHs* [7,19]. Seven putative *MDH* genes were isolated from *Stylosanthes guianensis*, suggesting the roles of *SgMDHs* in coping with nutrient and metal stresses [11]. Chen et al. identified 16 *MDH* genes in poplar via whole-genome sequence analysis and divided them into five subgroups with similar gene structures and conserved sequences [21]. Most of the candidate genes were significantly up-regulated in each tissue 25–30 days after salt stress. Twenty *MDH* genes were identified from the genome of the apple, among which one gene was highly expressed in the process of fruit development and flower bud differentiation [20]. A total of thirty and twenty-five *MDH* genes were identified in *Gossypium raimondii* and *Gossypium hirsutum*, respectively [22], and the expression pattern of the *GhMDH* gene under salt stress was analyzed. These studies provide reference resources for *MDH* genes of other crops, but the genome-wide *MDH* gene family in rice has not yet been identified and reported.

Through the association analysis of candidate genes realized via high-throughput sequencing, we can detect single nucleotide polymorphisms (SNPs) in order to ensure that the marker is located in the target region and tightly linked to the trait [23]. At present, some alleles and variational loci of many salt tolerance-related genes have been identified [24,25,26]. Shefali Mishra et al. performed the allelic linkage analysis of eight members of the HKT ion transporter gene family and found that the haplotypes of *HKT1:5* and *HKT2:3*, H5 and H1, were associated with salt tolerance, and a salt-tolerant allele was simultaneously identified in *HKT2* and *HKT1:5* [27]. The author also re-sequenced 21 salt stress related genes of different gene families and found that there was a significant correlation between these genes and salt tolerance, revealing that different gene families have different degrees of variation [28]. There are many favorable natural variations in natural germplasms, especially quantitative trait variations [29,30]. Mining these favorable natural variations will lay a foundation for breeding excellent varieties [31]. As one of the research hotspots in the exploration of the natural variation of rice, it is of great significance to use natural population-based candidate genes to study the natural variation of the rice *MDH* gene family and its relationship with salt tolerance through gene association analysis, which will be helpful to gain an in-depth understanding of the biological functions and mechanisms of salt tolerance-related genes and the genetic improvement of rice.

In this study, we identified 12 *MDH* genes in rice via bioinformatics methods, and analyzed their protein structure, phylogeny, cis-regulatory elements and gene expression pattern under salt stress. Additionally, the natural variation of the *OsMDH* gene family and its relationships with salt tolerance were evaluated by using a natural population of rice, and the favorable alleles of *OsMDH* key gene were identified through haplotype analysis.

## 2. Results

### 2.1. Identification and Characterization of MDH Genes in Rice

To comprehensively understand the evolutionary history of the rice *MDH* family and its importance under salt stress. We identified a total of 12 *MDH* members in the rice (*Oryza sativa* L.) genome, namely *OsMDH1*–*OsMDH12.1* (Table 1), which were unevenly distributed on rice chromosomes. There were two genes on chromosomes 1 and 8, respectively, and the remaining eight genes were distributed on chromosomes 2, 3, 4, 5, 6, 7, 10 and 12. The lengths of the *OsMDH* genes varied: *OsMDH6.1* was the shortest, with 1532 bp, whereas *OsMDH8.2* was the longest, with 5229 bp. The average lengths of their coding sequences and protein sequences were 1117 bp and 371.5 amino acids, respectively. The molecular weights of the OsMDH proteins ranged from 35.44 to 47.01 KDa, with an average of 39.43 KDa. The isoelectric points ranged from 5.97 to 9.03; an average of 7.82. A total of 12 *MDH* genes were predicted and located diversely in the nucleus, cytoplasm, and endoplasmic reticulum of rice. These results indicated that there were differences in the coding sequences and protein sequences among *MDH* genes, which may lead to different biological functions.

**Figure 1:**
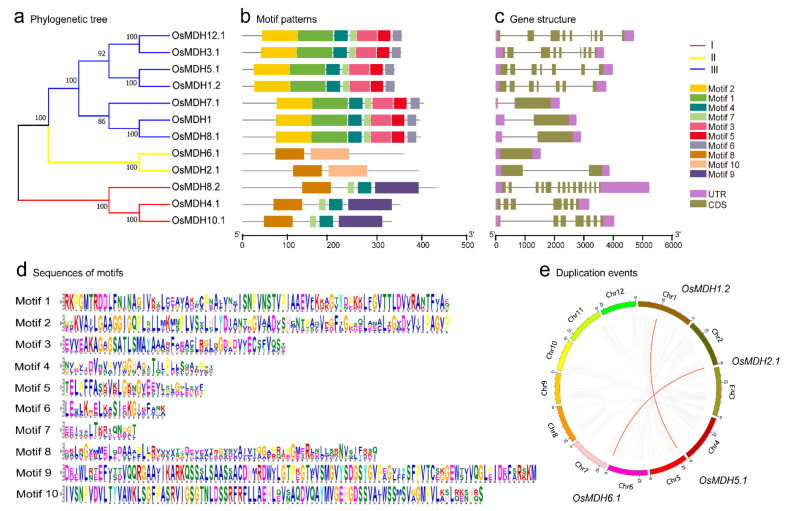
Phylogenetic relationships, gene structures and conserved motif analysis of *MDH* genes in rice. (**a**) The phylogenetic tree was constructed based on the full-length sequences of rice MDH proteins. (**b**) The distribution of conserved motifs in OsMDH; the ten different colored boxes represent ten different motifs. (**c**) Exon-intron structures of the *OsMDHs* genes. Green boxes indicate exons; black lines indicate introns, the upstream/downstream area is indicated by a purple box. (**d**) Sequence logo of the MDH proteins motifs. The height of each amino acid represents the relative frequency of the amino acid at that position. (**e**) Segmental duplication events of *MDH* genes in the *Oryza sativa* L. The gray curves indicate all the collinearity blocks in the rice genome, and the red curves indicate the segmental duplication events of *OsMDH* genes.

To explore the diversification and identify the phylogenetic relationships of MDH proteins in rice, we performed a MEME analysis and multiple sequence alignment with the 12 OsMDH amino acid sequences to build an unrooted phylogenetic tree. The results showed that the 12 OsMDHs could be divided into three subgroups, I, II, and III, according to the phylogenetic tree, motif type and quantity contained of each MDH protein (Figure 1a,b). A total of ten conserved motifs, designated as motif 1 to motif 10, were identified (Figure 1b,d). *OsMDH4.1*, *OsMDH10.1* and *OsMDH8.2* in group I had four types of motifs; *OsMDH6.1* and *OsMDH2.1* in group II had two types of motifs. Group III was the largest subgroup, with seven members containing seven types of motifs (Figure 1b). The *MDH* members within the same group contained the same motifs, and different groups contained different types and quantities of motifs. Motif 8 coexisted in groups I and II, and motifs 4 and 7 in opposite positions coexisted in groups I and III. Some conservative motifs existed only in one taxon, such as motifs 9 (group I) and 10 (group II). Conserved domains were predicted using the NCBI CDD program. Except OsMDH8.2, with the NAD (P) binding site, other amino acid sequences of MDH had common characteristics, including NAD binding, the dimerization interface and the substrate binding site. For example, motif 2 possessed the dinucleotide NAD binding domain with an important conserved glycine motif (GXXGXXG) (Figure 1d), which is a specific binding site of the NAD cofactor and is important in structure stabilization [22,32].

We explored the structural diversity of *MDH* genes to further understand their structural evolution. The exon–intron organization map analysis revealed that the number of exons varied from 1 to 14 (Figure 1c), the *MDH* genes in one subfamily or chromosome did not completely share similar gene structures in terms of either the intron/exon number or length. *MDH* members in group II contained the lowest number of exons, with an average of 1.5 exons. There were great differences in the number of exons of *MDH* members in group I. The *OsMDH8.2* in group I contained 14 exons, which is the maximum, whereas *OsMDH7.1*, *OsMDH8.1* and *OsMDH1* each contained only one exon. Overall, the distribution of the intron/exon number or length of *MDH* genes supported the clustering of the phylogenetic tree. These results indicated that the differences in evolution and function between *MDH* members are related to their motifs, and exon and intron differences.

Previous studies have shown that duplication events (segmental duplication, tandem duplication and transposition events) are the driving force behind genome evolution [33]. To further evaluate the relationship between the genetic divergence of the MDH gene family and gene duplication, segmental duplication and tandem duplication analysis were per-formed. We found two pairs of segmental duplications (*OsMDH1.2*/*OsMDH5.1* and *OsMDH2.1*/*OsMDH6.1*) that were derived from duplicated chromosomal regions of rice (Figure 1e), one of which belonged to group III, and the other one belonged to group II. In addition, the chromosome distribution analysis showed that tandem duplications had not been involved in the expansion of the rice *MDH* gene. These results indicated that the expansion of the *MDH* gene family in rice was mainly attributed to segmental duplication events.

### 2.2. Phylogenetic Analysis of MDH Proteins

**Figure 2:**
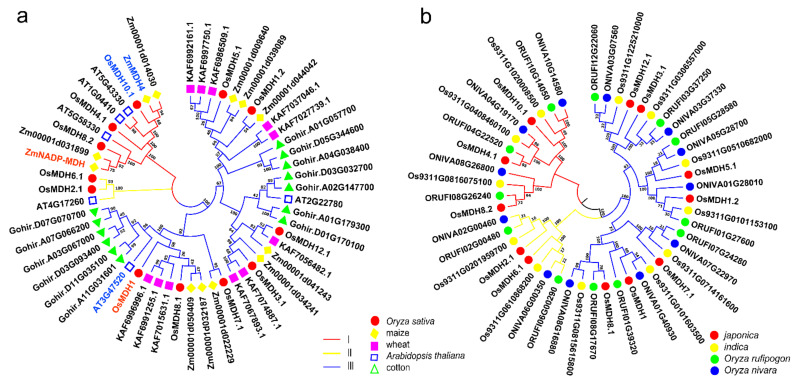
Phylogenetic tree of canonical *MDH* genes. (**a**) The phylogenetic tree was constructed by comparing the protein sequences of 54 *MDH* genes from five species, namely rice, maize, wheat, *Arabidopsis* and cotton. The red, yellow and blue branches represent groups I, II and III, respectively. Genes of rice are marked by red circles; genes of maize are marked by yellow triangles; genes of wheat are marked by pink squares; genes of *Arabidopsis* are marked by blue boxes; genes of cotton are marked by green triangles. A blue colored name indicates cloned genes associated with seed development, and a red colored name indicates cloned genes associated with salt response. (**b**) The phylogenetic tree was constructed by comparing the protein sequences of 48 *MDH* genes from *japonica*, *indica*, *Oryza rufipogon* and *Oryza nivara*. The red, yellow and blue branches represent groups I, II and III, respectively. Genes of *japonica* are marked by red circles; genes of *indica* are marked by yellow circles; genes of *Oryza rufipogon* are marked by green circles; genes of *Oryza nivara* are marked by blue circles. One thousand repeated boot values are displayed on each node, with the scale indicating the branch length.

In order to analyze the evolutionary relationships of the *MDH* gene family among different species, we constructed an unrooted phylogenetic tree using highly homologous protein sequences of another four species (6 homologous genes in *Arabidopsis*, 13 homologous genes in cotton, 12 homologous genes in maize and 11 homologous genes in wheat) and rice MDH family protein sequences. The results showed that 54 MDH protein sequences were divided into three groups (Figure 2a), which is consistent with the previous grouping results (Figure 1a). Group I contained homologous genes with *Arabidopsis* and *maize*, while group II contained only one *Arabidopsis* homologous gene. Group III contained the most *MDH* members, group III (three) had more branches than group I (two) and group II (one), and proteins were more phylogenetically divergent. The internal branches of group I and group III showed that the MDH proteins in rice were most homologous to maize and wheat, which may represent the evolutionary relationship between monocotyledons and dicotyledons and the conservation of MDH proteins in evolution. *OsMDH10.1* in group I was reported to be a key enzyme for starch synthesis in rice endosperm [9], and its homologous gene *ZmMDH4 (ZEAMMB73_Zm00001d032695)* regulates the balance between mitochondrial respiration, ATP production, and endosperm development [8], which indicates that homologous genes in different species may have similar functions. The *ZmNADP-MDH (X16084)* is known to be a salt tolerance gene [18], which has a closer evolutionary relationship with *OsMDH8.2*, and we inferred that *OsMDH8.2* may have a similar function to *ZmNADP-MDH.* In group III, *OsMDH1* was reported to play a negative role in salt tolerance through the regulation of vitamin B6 content in rice tissues [12], *OsMDH8.1* and *OsMDH1* were on the same evolutionary branch, which is a paralogous gene pair. The relationship between the *OsMDH8.1* gene and salt stress response warrants further verification.

At the same time, we performed phylogenetic analysis of the *MDH* gene family to analyze the evolutionary relationships in *Oryza*. We identified the *MDH* gene family of the Nipponbare, which belongs to the *japonica* rice subgroup, two wild rice ancestors (*Oryza rufipogon* and *Oryza nivara*) [34] and an *Indica* rice variety (93-11) [35] at the whole-genome level (Figure 2b). All the rice species contained 12 *MDH* genes, respectively. The phylogenetic tree revealed that all the MDH proteins in the various rice species clustered into three major groups, similar to the above clustering results (Figure 1a). There were different conserved domains between each subgroup. The *MDH* gene family in cultivated rice originated from its wild relatives. The number of *MDH* gene families in *Oryza* was unchanged in quantity, which showed that the *MDH* family did not expand or shrink, and the *MDH* gene family in *Oryza* was quite conserved during the long-term evolutionary process.

### 2.3. Putative Cis-Regulatory Elements Analysis in the Promoter of OsMDH Genes

**Figure 3:**
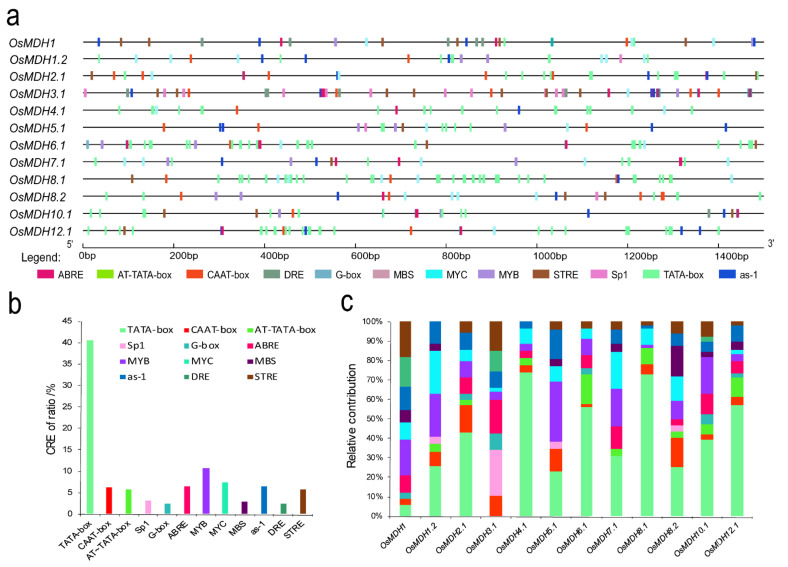
Putative regulatory *cis*-elements of *OsMDH* gene promoters. (**a**) The relative positions of cis-regulatory elements are shown on the line representing the 1500 bp upstream region of each *OsMDH* gene promoter. Only *cis*-elements required for MBS, G-box, DRE, Sp1, AT-TATA-box, STRE, CAAT-BOX, ABRE, as-1, MYC, MYB, and TATA-BOX are shown. (**b**) Percentage distribution of *cis*-regulatory elements in the promoters of *OsMDH* genes. (**c**) The distribution of various elements in the promoter regions of *OsMDH* genes are shown by different colors.

The analysis of *cis*-regulatory elements (CREs) in the promoter region is essential for the elucidation of the gene expression pattern [36]. We submitted the promoter regions of 12 *MDH* gene with length of 1500 bp to the online tool PlantCARE to analyze the *cis*-acting elements. The type and position of *cis*-elements were marked by different colors (Figure 3a). A total of twelve putative CREs, namely MBS, G-box, DRE, Sp1, AT-TATA-Box, STRE, CAAT-Box, ABRE, as-1, MYC, MYB and TATA-box, were predicted to have the most probability. The proportion of core promoter element (TATA-box) was the highest (41%), and almost all genes contained a large number of core promoter elements (except *OsMDH3.1*). This was followed by MYB (11%), MYC (7%), ABRE (6.6%), as-1 (6.6%), STRE (5.7%), CAAT-box (6.3%) and AT-TATA-box (5.7%). Sp1(3.1%), G-box (2.4%), MBS (2.8%) and DRE (2.4%) also had small percentages (Figure 3b). In addition to *OsMDH1.2*, *OsMDH5.1* and *OsMDH8.1*, all the other genes had ABRE response elements. Moreover, most promoters contained defense and stress response elements, including drought-responsive elements and salt-responsiveness elements, such as MYB, MYC, DRE, STRE, as-1 and MBS (Figure 3c). These elements exist in promoters of maize and rice resistance genes or transcription factors, and in many genes are related to the regulation of abiotic stress [37,38,39,40]. The promoters of four and six *OsMDH* genes contained Sp1 and G-box elements involved in the photo response, respectively. These results indicated that the relevant CREs will respond to the expression of the *MDH* genes and improve plant resistance under abiotic stress.

### 2.4. Expression Pattern of MDH Genes in Rice

**Figure 4:**
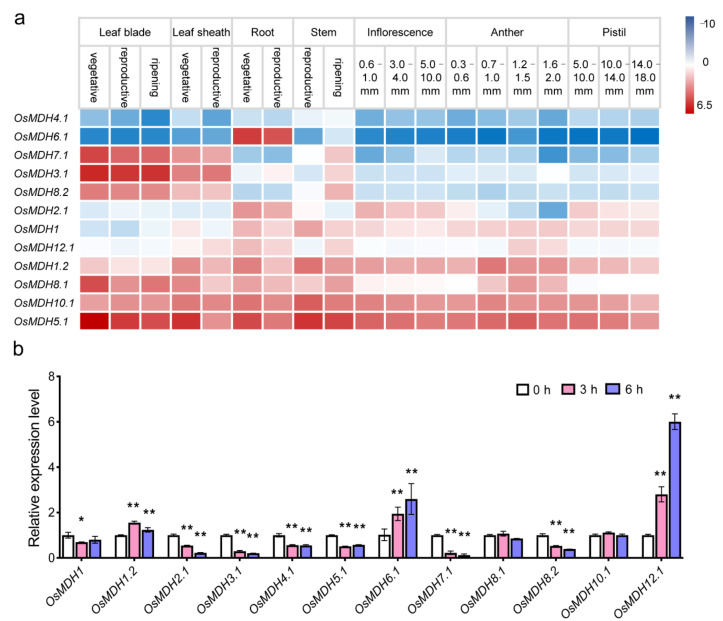
Expression patterns of *OsMDH* gene family in rice. (**a**) The expression profiles of different tissues and development stages of *OsMDH* genes in rice without salt treatment. (**b**) Expression analysis of 12 *OsMDH* genes under salt stress by qRT-PCR. * and ** indicate a significant difference between the treatment and control at the 0.05 and 0.01 probability levels, respectively.

In order to understand the role of *OsMDH* genes during different development stages in rice, *OsMDH* expression data were downloaded from the RiceXPpro V3 database, including the gene expression profiles of different tissues at both vegetative and reproductive stages (Figure 4a). *OsMDH1.2*, *OsMDH5.1* and *OsMDH10.1* (*FLO16*) were constitutively expressed at a high level in all tissues and all the development stages, whereas the expression of *OsMDH4.1* was relatively lower. *OsMDH3.1*, *OsMDH7.1* and *OsMDH8.2* were highly expressed in leaves, leaf sheaths and stems, and the *OsMDH6.1* was specifically expressed in roots.

To verify whether other genes of the *OsMDH* family are also related to salt stress, we performed qRT-PCR to analyze the expression level of 12 candidate genes after salt treatment for 3 h and 6 h, respectively (Figure 4b). The results showed that the expression levels of the ten remaining genes significantly changed, except for *OsMDH10.1* and *OsMDH8.1*. *OsMDH1.2*, *OsMDH6.1* and *OsMDH12.1* were significantly upregulated under salt stress, which may positively regulate salt tolerance in rice. In addition, *OsMDH1*, *OsMDH2.1*, *OsMDH3.1*, *OsMDH4.1*, *OsMDH5.1*, *OsMDH7.1* and *OsMDH8.2* were significantly downregulated, which may play a negative role in resistance to salt stress. The expression of *OsMDH1* was significantly downregulated after treatment for 3 h and played a negative role in rice salt tolerance, which was consistent with previous studies [12]. However, there was no significant difference in the expression after 6 h salt treatment, which may be related to the different expression levels at different degrees of salt stress treatment. Notably, compared with the expression level before salt stress, the expression of *OsMDH12.1* was three times higher at 3 h after salt treatment, and then reached six times higher at 6 h, indicating that *OsMDH12.1* could be an important candidate gene involved in salt stress response. These results indicated that more than 83% of the *OsMDH* genes are involved in salt stress response. Combined with *cis*-regulatory element analysis, we speculated that most of the *OsMDH* genes were involved in salt stress response through *cis*-regulatory elements related to plant hormones and abiotic stress response.

### 2.5. Gene-Based Association and Haplotype Analysis of OsMDH Genes

In order to clarify whether these *MDH* genes are related to salt tolerance and further investigate their natural variations, we used 609 natural populations to conduct a candidate gene association analysis [29]. The SNPs in the CDS region and 2.5 kb upstream of the initiation codon (ATG) of each *MDH* gene were extracted, respectively. After screening with the parameters, 557 high-quality SNPs were obtained. We found that all 12 *MDH* genes contained polymorphic loci, with an average of 46.42 SNPs per gene. *OsMDH12.1* had the greatest number of polymorphic loci with 159 SNPs (Table 2).

Combined with the phenotypic identification of salt tolerance levels of 664 cultivars at the seedling stage [29], the general linear model (GLM) and compressed mixed linear model (CMLM) were used to identify the association between the traits and SNPs of *MDH* genes. *p*-values corresponding to all SNPs in the regions of 12 *MDH* genes were statistically analyzed (Table 2). In the GLM model, 6, 14, 6, 4 and 134 SNPs were detected for *OsMDH4.1*, *OsMDH6.1*, *OsMDH8.1*, *OsMDH8.2* and *OsMDH12.1*, respectively, which were significantly correlated with salt tolerance (*p* ≤ 0.01). In addition, four and three SNPs of *OsMDH8.1* and *OsMDH12.1*, respectively, were significantly correlated with salt tolerance (*p* ≤ 0.001). In the CMLM model, two SNPs (*p* ≤ 0.01) of *OsMDH8.1* were detected in the promoter and were significantly correlated with salt tolerance (Table 2). *OsMDH8.1* and *OsMDH12.1* had a large number of natural variation sites that were significantly related to salt tolerance under GLM (*p* ≤ 0.001). Therefore, we focused on the two genes in the follow-up study.

**Figure 5:**
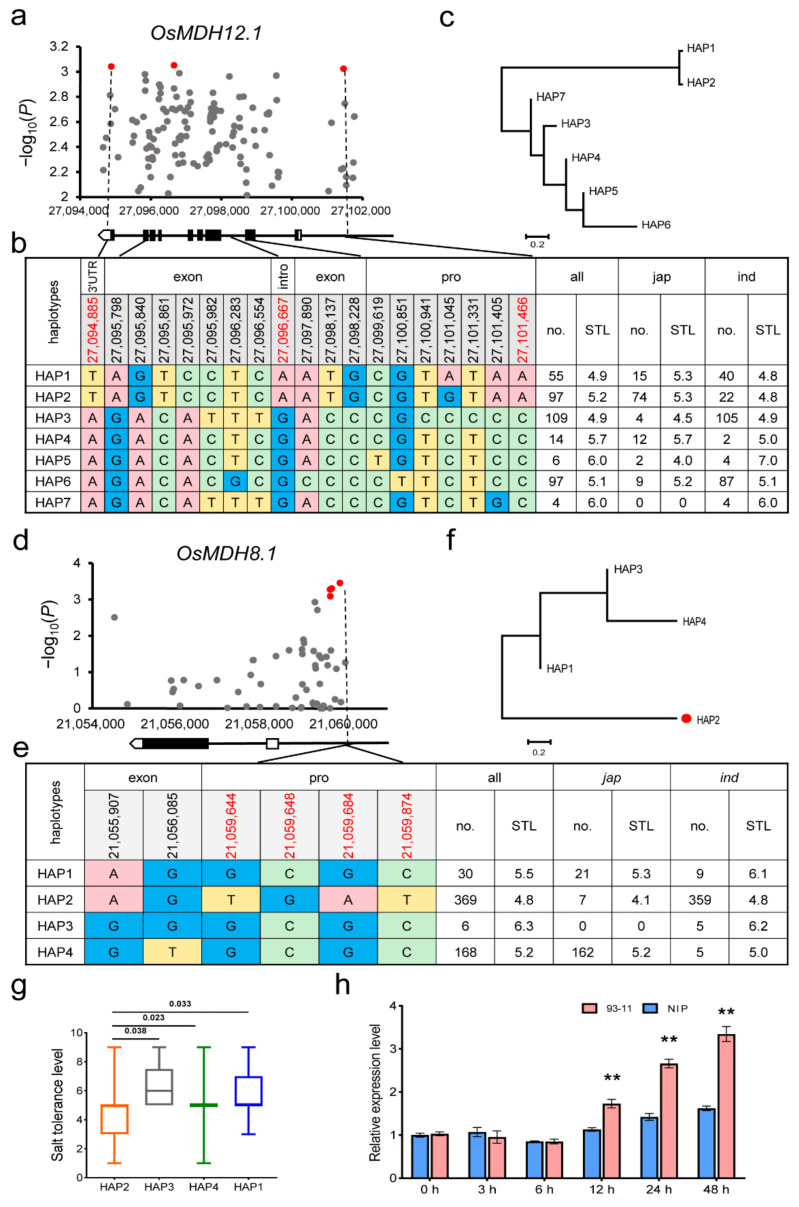
Association analysis and haplotype analysis of *OsMDH12.1* and *OsMDH8.1* with rice salt tolerance. (**a**) Red dots represent significant SNPs detected in *OsMDH12.1* related to salt tolerance level, and a gene structure diagram is shown below it. (**b**) Seven *OsMDH12.1* haplotypes and their distribution in *indica* and *japonica*. The location of significant SNPs is indicated in red. (**c**) Phylogenetic tree for *OsMDH12.1* haplotypes developed by MEGA 7. (**d**) Red dots represent significant SNPs detected in *OsMDH8.1* related to salt tolerance level, and a gene structure diagram is shown below it. (**e**) Four *OsMDH8.1* haplotypes and their distribution in *indica* and *japonica*. The location of significant SNPs are indicated in red. (**f**) Phylogenetic tree for *OsMDH8.1* haplotypes developed by MEGA 7 with all the non-synonymous SNPs and significant SNPs. HAP2 is represented by red dots. (**g**) Comparison of salt tolerance level (STL) of *OsMDH8.1* haplotype (**h**) Relative *OsMDH8.1* expression level of the 93-11 (HAP2) and NIP (HAP4) in 0–48 h by salt stress. ** indicated significant difference (*p* < 0.01) by student’s *t* test. 93-11 indicates *Indica* rice variety 93-11; NIP indicates *Japonica* variety Nipponbare; h indicates hours.

To further explore natural variations of *OsMDH8.1* and *OsMDH12.1* in germplasms, we performed haplotype analysis for *OsMDH8.1* and *OsMDH12.1* using 609 rice accessions. We found three significant SNPs (*p* < 0.001) (27,094,885, 27,096,667 and 27,101,466) in the promoter, intron and 3’UTR, respectively (Figure 5a). Through investigating the haplotypes of *OsMDH12.1* in 609 germplasms, we found seven haplotypes (Figure 5b). Phylogenetic analysis showed that the haplotypes of *OsMDH12.1* were divided into two clusters (Figure 5c). HAP1 and HAP2 clustered into one clade, accounting for 60.2% of *japonica*. HAP3-HAP7 was clustered into another clade, accounting for 88.8% of the *indica* genotype. HAP3-HAP6 probably evolved from HAP7 through continuous selection. By comparing the salt tolerance phenotypes among different haplotypes, it was found that there were no significant differences among the seven haplotypes (Figure 5b). Although *OsMDH12.1* had significant variation in loci in the natural population, no functional haplotype was found.

In *OsMDH8.1*, four significant SNPs (21,059,644, 21,059,648, 21,059,684, 21,059,874) in the promoter were associated with the salt tolerance grade in all the populations under GLM (*p* ≤ 0.001) (Figure 5d). There were four significant SNPs in the promoter and two non-synonymous sites in the CDS region of *OsMDH8.1* that were used for the genotypic classification of 573 varieties (Figure 5e). The results showed that HAP2 was the most prevalent allele in *indica* and HAP4 was the main allele in *japonica* subpopulations. These haplotypes were clustered into two clades (Figure 5f). By comparing the salt tolerance phenotypes of different haplotypes, we found that the salt tolerance level of HAP2 was significantly lower than that of *HAP1*, HAP3 and HAP4 (Figure 5g). This suggests that HAP2 is the elite haplotype for salt tolerance in rice and the natural variations in the promoter of *OsMDH8.1* should be important for its function. For further verification, the *indica* rice variety 93-11 (HAP2) and *japonica* representative rice Nipponbare (HAP4) were used for the expression analysis of *OsMDH8.1* under salt stress. The expression of 93-11 (HAP2) was significantly higher than that of Nipponbare at 12 h and 48 h after salt stress treatment, but no difference was shown under normal conditions or slight salt stress (Figure 5h), suggesting that the four SNPs sites in the *OsMDH8.1* promoter may be essential for its induced expression under severe salt stress.

## 3. Discussion

Salt tolerance is known to be the result of multi-gene interactions [41,42]. The morphological variation caused by salt stress is related to the metabolic process [13,16,43,44]. Malate dehydrogenase (MDH) is a highly active enzyme in plants, an increasing number of *MDH* genes have been identified in different species, which play an important role in responding to abiotic stress [4,5,21,45]. At present, only two members of the rice *MDH* family have been reported [9,12], which are related to rice development and salt tolerance, but we still know little about the functions of other members of the *MDH* family in the model plant for rice. The development of genome sequencing technology and extensive expression profile data allow us to study the response of the *MDH* family to salt stress.

In this study, we identified 12 *MDH* genes in the rice genome, which is less than the number of genes identified in diploid *G. raimondii* (13), tetraploid *G. hirsutum* (25) [22] and *P. trichocarpa* (16) [21], which is more than the number identified in diploid *P. vulgaris* (8) [45]. All *OsMDH* genes have different subcellular localizations (cytoplasmic, endoplasmic reticulum, and nuclear), which may result in differences in function. Comparative analysis of *MDH* families from different plant species revealed that the *MDH* family has experienced extensive expansion during evolution. Based on the phylogenetic tree, *MDH* family genes could be divided into groups I, II and III in both monocotyledons (rice and wheat) and dicotyledons (*Arabidopsis* and cotton), which indicated that the *MDH* family is relatively conserved during the long-term evolutionary selection process. We found the homologous pair formed by eudicot and monocot in the phylogenetic tree, indicating that before the eudicot–monocot split, a common ancestral *MDH* gene existed. Within the group, *MDH* subfamilies contained the same type and numbers of motifs, we speculated that the *MDH* members in each subfamily may have similar functions.

Recent studies have proposed that gene duplication is considered to be one of the primary driving forces in the expansion of gene families and genome evolution [35,46]. Furthermore, we found that *OsMDH* genes were unevenly distributed across ten of the twelve chromosomes in rice. We identified two segmental duplication events involving four genes but no tandem duplication events, which indicated that the segmental duplication events contributed to the evolution of *MDH* genes in the rice genome. Previous studies found that there are both fragment repetition and tandem repetition events to expand gene families in cotton [22] and poplar [21], which is inconsistent with the research in rice, we conjectured that this may be related to the differentiation of species and their genome sizes.

Gene expression profiling and qRT-PCR can help to reveal the expression pattern of *MDH* families in rice. The expression of *MDH* genes showed obvious tissue specificity. qRT-PCR results showed different expression patterns of *MDH* genes under salt stress. We found that three genes showed an up-regulation trend, and seven genes showed a down-regulation trend under salt stress, which indicated that there may be different tolerance mechanisms in rice. The change in *MDH* gene expression may be closely related to the *cis*-regulatory elements in the promoter region. It is reported that *SnRK2s* can phosphorylate various ABRE binding proteins, AREB and ABF transcription factors, and further regulate ROS scavenging, ion homeostasis and stomatal closure in response to salt stress [47,48,49]. ABREs *cis*-acting elements can also bind to bZIP transcription factors to regulate downstream gene expression and improve the salt tolerance of plants [50,51]. DRE elements are commonly found in promoters of genes responding to drought, salt and low-temperature stress; it can combine with DREB transcription factors specifically to enhance the stress tolerance of plants [52]. In this study, hormone-responsive elements (ABREs), drought response (DRE, MYC, MYS, and MYB recognition sites) and osmotic stress response (STRE) accounted for a major proportion of the *MDH* family promoter *cis*-acting elements, which may play important roles in regulating the response of *MDH* genes to abiotic stress. These results indicate that the *MDH* genes plays a crucial role in the response of rice to salt stress and can be considered as a candidate gene for further research on the molecular mechanisms of salt tolerance.

In recent years, the association analysis of candidate genes based on whole genome sequencing has been widely used in maize [52], rice [53], wheat [54], *Arabidopsis* [55], cucumber [56] and other crops and vegetables to mine favorable alleles based on natural variations, narrow the target range and identify target genes more effectively and accurately. In addition to analyzing the function of a single gene in a single genotype, this method has also been gradually applied to analyze different candidate genes or gene families [27,53,57,58]. Liu et al. identified the association between the natural variation of the *ZmDREB* gene family and the drought tolerance of maize at the seedling stage; a beneficial *ZmDREB2.7* allele was effective in improving tolerance to drought stress [52]. In this study, the relationship between natural variation and salt tolerance phenotypes in 12 genes of the rice *MDH* family was determined. A total of two *OsMDH* genes (*OsMDH8.1* and *OsMDH12.1*) were found to have significant (*p* < 0.001) SNPs related to salt tolerance levels at the seedling stage (Table 2). It has been reported that *OsMDH1* plays a key role in the ROS detoxification process induced by sodium chloride [12]. However, we did not find natural variants that were significantly associated with the salt-tolerance phenotype at the seedling stage. Although the *OsMDH12.1* gene was upregulated by induction and the expression level was the most significant under salt stress, we did not identify the dominant haplotype related to salt tolerance. These results may be caused by the different genetic effects of different genes or may be related to the complexity of salt tolerance traits and population materials. Interestingly, combined with haplotype analysis and expression verification, we speculated that the natural variation of *OsMDH8.1* contributed to the salt stress tolerance of rice seedlings. Polymorphism in the promoter region may be the functional variation that leads to the gene expression difference and salt tolerance in rice; causal SNPs require further verification through molecular biological analysis.

## 4. Materials and Methods

### 4.1. Identification of the MDH Gene Family in Rice

The whole genome, protein sequences and GFF3 gene annotation files of Nipponhare, 9311, *Oryza rufipogon* and *Oryza nivara* were downloaded from the database: *japonica* variety Nipponhare (RGAP1.0 database, http://rice.uga.edu/pub/data/Eukaryotic_Projects/o_sativa/annotation_dbs/pseudomolecules/version_7.0/all.dir/ (accessed on 22 October 2021)), *indica* variety 9311 [34] (http://ricerc.sicau.edu.cn/RiceRC/download/downloadBefore (accessed on 22 October 2021)), *Oryza rufipogon* and *Oryza nivara* (http://plants.ensembl.org/info/data/ftp/index.html (accessed on 22 October 2021)) [33]. Protein sequences were constructed using the local BLAST database. The protein sequences of the *MDH* gene family in *Arabidopsis* were obtained from the UniProt database (https://www.uniprot.org/ (accessed on 22 October 2021)), and the *MDH* gene family in rice were screened using local BLAST (E < 1 × 10^−10^, Identity > 40%). Then, a reliable *MDH* gene family list was obtained from SMART (http://smart.embl.de/ (accessed on 22 October 2021)), Pfam and NCBI CDD conservative domain databases; we built an *MDH* gene family Hidden Markov Model (HMM) to identify the *MDH* gene in the other three genomes.

The physical and chemical properties of *MDH* gene families in rice, such as the molecular weight and isoelectric point, were predicted using the online tool ExPASy (http://web.expasy.org/protparam/ (accessed on 22 October 2021)). Sub-cellular localization prediction was performed using the online tool Psort (https://www.genscript.com/psort.html (accessed on 22 October 2021)). The candidate genes were named according to their chromosome location.

### 4.2. Construction of Phylogenetic Tree of the MDH Gene Family

The homologous sequence alignment of candidate genes was carried out using the NCBI website (https://blast.ncbi.nlm.nih.gov/Blast.cgi (accessed on 18 March 2022)), and the amino acid sequence of homologous genes in *Zea mays*, *Triticum aestivum*, *Gossypium hirsutum* and *Arabidopsis thaliana* (E < 1 × 10^−10^, identity > 70%) were downloaded. The multiple sequence alignment of MDH protein sequences was performed using ClustalW in MEGA 7.0.26 with the default parameters. A neighbor-joining phylogenetic tree of *Oryza sativa* L., *Zea mays*, *Triticum aestivum*, *Arabidopsis thaliana* and *Gossypium hirsutum* was constructed based on the alignment results of the Poisson model, pairwise deletion and 1000 bootstrap replications. The phylogenetic tree of MDH amino acid sequences of Nipponhare, *indica* variety 93-11, *Oryza rufipogon* and *Oryza nivara* was constructed in the same way.

### 4.3. Structure and Motif Analysis of the MDH Gene Family in Rice

MEME online software (http://meme-suite.org/tools/meme (accessed on 22 October 2021)) was used to analyze the motifs of MDH protein sequences, and the parameters were set as follows: amino acid length: 6–100; number of repeats of the motif: arbitrary; threshold number of motif discovery: 10. TBtools was used to analyze all the genetic structure of the *MDH* genes. To analyze the promoter, 1500 bp genomic DNA sequences upstream of the initiation codon (ATG) of each *MDH* gene were extracted from the genome database. Then, the *cis*-regulatory elements of each promoter sequence were predicted using the PlantCARE database (http://bioinformatics.psb.ugent.be/webtools/plantcare/html/ (accessed on 22 October 2021)), and the promoter structure and motif were visualized using TBtools.

### 4.4. Duplication Analysis of OsMDH

*MDH* gene duplication events were analyzed using the Multiple Collinearity Scan toolkit (MCScanX version 0.8). Circos-0.69–6 was used to map the results of the *MDH* gene duplication events as images.

### 4.5. Plant Materials and Salt Stress Treatment

Nipponbare and 93-11 were used in this experiment. The seeds were germinated and soaked in culture solution at 28 °C for 14 days. After that, the seedlings were transferred to 150 mmol/L NaCl solution. Leaf samples were collected at 0 h, 3 h, 6 h, 9 h, 12 h, 24 h and 48 h, respectively. The collected samples were immediately frozen in liquid nitrogen and stored at −80 °C.

### 4.6. Transcriptomic Analysis, RNA Extraction and qRT-PCR Analysis

The expression level of *OsMDH* gene family members in different organs and developmental stages were downloaded from the RiceXPpro V3 database (https://ricexpro.dna.affrc.go.jp/ (accessed on 22 October 2021)). A gene expression heat map was generated using Office 2016 software.

Total RNA was extracted from tissue samples collected at different time points using TRIzol (Invitrogen, Carlsbad, CA, USA, Cat. AM1912), and Dnase I was added to remove DNA. cDNA was synthesized via reverse transcription using the SuperScript first-stand Synthesis SuperMix (Thermo, Waltham, MA, USA,) kit. qRT-PCR was performed using an ABI 7500 fast real-time PCR system (Table 3), and the ubiquitin gene was used as an internal reference [59]. The reaction was carried out as follows: 95 °C for 5 min, followed by 40 cycles of 95 °C for 5 s, 58 °C for 30 s, and 70 °C for 30 s. Each reaction was performed using three biological replicates, and the expression levels of the genes were calculated using the 2^−ΔΔC*t*^ method. Microsoft Excel 2016 was used for data processing, and GraphPad Prism 8.0 software was used for construction.

### 4.7. Association Analysis of Candidate OsMDH Genes

The physical positions of 12 *OsMDH* genes were obtained from the online database (https://www.ricedata.cn/ (accessed on 22 October 2021)). All SNPs of the 12 *MDH* genes (including 2.5 kb promoter region and coding sequence) were obtained from 3K rice sequencing data (https://snp-seek.irri.org/ (accessed on 22 October 2021)), and the parameters were set as follows: the minimum allele frequency (maf) was greater than 0.05, and the miss rate was less than 30%. A total of 557 high-quality SNPs were obtained. The salt tolerance level of 609 rice materials after 7 days of 0.9% NaCl stress at the seedling stage were obtained from previous studies [29]. Gene-based association analysis was performed according to a GLM and CMLM using the TASSEL 5.0 program (significant threshold *p* = 0.01) [60].

### 4.8. Haplotype Analysis of Candidate Genes

The SNPs with a significant correlation in the promoter, or non-synonymous variations in the coding sequence, were used for haplotype analysis. After classification, the haplotypes with less than three accessions were manually removed. The significance of phenotypic variation among different haplotypes was calculated through analysis of variance (ANOVA) with Duncan’s multiple range test using SPSS software. The haplotypes were sorted into Hapmap format and converted into phylip format using TASSEL5 [60]. The phylip file was imported into MEGA 7.0, and a *.meg file was obtained. The linear neighbor joining (NJ) tree was constructed at 10,000 bootstrap values by MEGA 7.0.

## 5. Conclusions

In this study, we found that: (1) 12 *MDH* genes were identified in the whole genome of rice, which were closely related to other monocotyledons; (2) 12 *OsMDH* genes were divided into three groups, and the genes in the same group had similar gene structures; (3) promoter *cis*-regulatory element analysis and salt stress-induced expression analysis indicated that most *OsMDH* family genes were involved in salt stress response; (4) the association analysis of candidate genes confirmed that natural variations existed in two *OsMDH* genes and were significantly correlated with salt stress; (5) the functional variation of *OsMDH8.1* in the promoter may play a critical role in the differences in gene expression and salt tolerance; (6) *OsMDH8.1* and its favorable alleles may be important genetic resources for the genetic improvement of salt tolerance in rice.

## Figures and Tables

**Table 1:** Basic information of *MDH* gene family in rice.

Gene ID	Gene Name	Chr	Start	End	Genomic Sequence Length (bp)	CDS (bp)	Protein Length (aa)	MW (kDa)	Isoelectric Point (PI)	Subcellular Localization
LOC_Os01g61380	*OsMDH1* [12]	1	35499017	35501765	2749	1191	396	41.79	7.90	cytoplasmic
LOC_Os01g46070	*OsMDH1.2*	1	26190752	26194517	3766	1023	340	35.46	8.74	cytoplasmic
LOC_Os02g01510	*OsMDH2.1*	2	295302	299174	3873	1179	392	42.72	7.29	endoplasmic reticulum
LOC_Os03g56280	*OsMDH3.1*	3	32086001	32089685	3685	1065	354	37.02	8.06	cytoplasmic
LOC_Os04g46560	*OsMDH4.1*	4	27605166	27608347	3182	1059	352	38.30	7.22	endoplasmic reticulum
LOC_Os05g49880	*OsMDH5.1*	5	28617595	28621585	3991	1023	340	35.44	8.30	nuclear
LOC_Os06g01590	*OsMDH6.1*	6	346985	348516	1532	1083	360	38.72	8.46	nuclear
LOC_Os07g43700	*OsMDH7.1*	7	26153825	26156006	2182	1215	404	42.22	9.03	nuclear
LOC_Os08g33720	*OsMDH8.1*	8	21054659	21057561	2903	1194	397	41.54	7.54	cytoplasmic
LOC_Os08g44810	*OsMDH8.2*	8	28141042	28146270	5229	1302	433	47.01	7.34	endoplasmic reticulum
LOC_Os10g33800	*OsMDH10.1(FLO16)* [9]	10	17913818	17917850	4033	999	332	35.57	5.97	endoplasmic reticulum
LOC_Os12g43630	*OsMDH12.1*	12	27094647	27099351	4705	1071	356	37.39	7.99	cytoplasmic

**Table 2:** Association analysis of natural variation in *OsMDH* genes with salt tolerance at the seedling stage in the rice diversity panel.

Gene ID	Gene Name	Polymorphic Number	GLM (*p* ≤ 0.01)	GLM (*p* ≤ 0.001)	CMLM (*p* ≤ 0.01)	CMLM (*p* ≤ 0.001)
LOC_Os01g61380	*OsMDH1*	9	0	0	0	0
LOC_Os01g46070	*OsMDH1.2*	9	0	0	0	0
LOC_Os02g01510	*OsMDH2.1*	19	0	0	0	0
LOC_Os03g56280	*OsMDH3.1*	40	0	0	0	0
LOC_Os04g46560	*OsMDH4.1*	63	6	0	0	0
LOC_Os05g49880	*OsMDH5.1*	29	0	0	0	0
LOC_Os06g01590	*OsMDH6.1*	49	14	0	0	0
LOC_Os07g43700	*OsMDH7.1*	33	0	0	0	0
LOC_Os08g33720	*OsMDH8.1*	56	6	4	2	0
LOC_Os08g44810	*OsMDH8.2*	35	4	0	0	0
LOC_Os10g33800	*OsMDH10.1*	56	0	0	0	0
LOC_Os12g43630	*OsMDH12.1*	159	134	3	0	0

**Table 3:** Primer sequence for qRT-PCR and gene annotation information.

Gene ID	Primer Name	Sequence of Forward Primer	Sequence of Reverse Primer	Annotation
LOC_Os01g61380	*OsMDH1*	CGAAAGCTGGTGCTGGATCTG	CACGGAGGGATGACTCAACA	*OsMDH1* [12]
LOC_Os01g46070	*OsMDH1.2*	AACGCCGGCATCGTTAAGAAC	GGGTTGCTGATCATGTTGACAAG	*lactate/malate dehydrogenase*
LOC_Os02g01510	*OsMDH2.1*	CGGCACCAACCTCGACTC	CGTGCTCTCCCACCATGTAC	*lactate/malate dehydrogenase*
LOC_Os03g56280	*OsMDH3.1*	TGGCGTTGTGGAATGTTCA	GGCTCCAGCACGACCTAAC	*lactate/malate dehydrogenase*
LOC_Os04g46560	*OsMDH4.1*	CGAGGCTGAGGCGTTCAAG	GCAGAGGCCTGGGATTTGTAG	*lactate/malate dehydrogenase*
LOC_Os05g49880	*OsMDH5.1*	GCCAGCTTTCCGAGTTTGAGAAG	GTTCGCGTGAGCAAACTTGATG	*lactate/malate dehydrogenase*
LOC_Os06g01590	*OsMDH6.1*	AGCGCGTACGAGGTGATCAAG	GATGCTGGCGACGGAGTAG	*lactate/malate dehydrogenase*
LOC_Os07g43700	*OsMDH7.1*	GCGCTGCACCTGTACGAC	CGTGTTGCAGTGTCCAAGATC	*lactate/malate dehydrogenase*
LOC_Os08g44810	*OsMDH8.1*	GCAGAGGACATCGTGTTCAGTA	CGTCCATTGCCACATCTTTAACTAG	*lactate/malate dehydrogenase*
LOC_Os08g33720	*OsMDH8.2*	GCTGACCTTGAGGGAGTGA	TCGATGCCCTTCTCGATACTG	*lactate/malate dehydrogenase*
LOC_Os10g33800	*OsMDH10.1*	AGCAAACACCAACGCTCTCATC	TGCCCTGTTGTGGTCAAGA	*FLO16* [9]
LOC_Os12g436301	*OsMDH12.1*	GCCAGCCACAGTTGGAAA	CCCAGGCTTACGAGGAACA	*lactate/malate dehydrogenase*
LOC_Os03g13170	*Ubi*	AACCAGCTGAGGCCCAAGA	ACGATTGATTTAACCAGTCCATGA	*Ubq (ubiquitin fusion protein*) [60]

## Data Availability

All data in the present study are available in the public database, as mentioned in the Section 4.
